# Chronic actinic dermatitis: a retrospective study of epicutaneous and photo epicutaneous tests between 2007‒2023^☆^

**DOI:** 10.1016/j.abd.2024.03.011

**Published:** 2024-11-13

**Authors:** Maria Antonieta Rios Scherrer, Mariana David Cangussu Fernandes Ribeiro, Hannah Barbosa Lopes dos Anjos, Vanessa Barreto Rocha

**Affiliations:** a Dermatology Annex, Hospital das Clínicas da Universidade Federal de Minas Gerais, Belo Horizonte, MG, Brazil; b Department of Internal Medicine, Faculty of Medicine, Universidade Federal de Minas Gerais, Belo Horizonte, MG, Brazil

Dear Editor,

Chronic actinic dermatitis (CAD) is a photodermatosis of uncertain etiology, and it has been proposed it is due to increased susceptibility to delayed hypersensitivity reactions to endogenous and exogenous photoallergens.^1^, ^2^ It is characterized by eczema in photoexposed areas, sparing photoprotected regions. It is diagnosed by the clinical picture, by histopathological changes compatible with eczema, and by decrease in the minimum erythematous dose (MED) in phototests with UVB and UVA in most cases.^3^

Of the 1,920 patients submitted to epicutaneous tests at the contact dermatitis outpatient clinic in Hospital das Clínicas, UFMG, from 2007 to 2023, 37 (1.9%) underwent photo epicutaneous tests, and 21 (1.1%) were diagnosed with CAD based on the clinical picture or by presenting lesions triggered or worsened by sun exposure. Patients using photosensitizing medications, those with lupus erythematosus, other collagen diseases, rosacea, and other related conditions were excluded. Patch tests were performed using the current Brazilian standard battery (FDA Allergenic RJ, Brazil) placed in Finn Chambers® with Scanpor® tape (Smartpractice, USA) or Allergochambers® (Neoflex, São Paulo), according to published techniques.^4^ Weeks later, at variable time periods, photo epicutaneous tests were performed with allergens from FDA Allergenic (RJ, Brazil) and Chemotechnique Diagnostics (Sweden) according to published references.^5^ (Table 1). The phototest was performed at the same time, by irradiating each gluteal region with UVB (Handisol®, National Biologic Corporation model Sel 002) and UVA (Prolumina, SP) light, using regularly calibrated devices. UVB doses were calculated according to the phototype (Table 2), and for UVA, 5 and 10 J/cm^2^ were tested. Readings were taken immediately after the emission of the lights, 30 and 60 min, and 24 h and 48 h after the irradiation. Since no patient showed MED less than 10 J/cm^2^ for UVA, the set of allergens was irradiated with this dose.

**Table 1:** Battery of photo epicutaneous tests used in the service.

Nº	Substance	Concentration	Vehicle
1	Octyl dimethyl PABA	5%	Solid petroleum jelly
2	Oxybenzone	3%	Solid petroleum jelly
3	Butyl methoxydibenzoylmethane	10%	Solid petroleum jelly
4	Sesquiterpene lactone mix (costunolide, allantolactone, dehydrocostus lactone)	0,1%	Solid petroleum jelly
5	Musk xylene	1%	Solid petroleum jelly
6	Balsam of Peru	25%	Solid petroleum jelly
7	Promethazine	1%	Solid petroleum jelly
8	Perfume mix	7%	Solid petroleum jelly
9	Irgasan	1%	Solid petroleum jelly
10	Potassium bichromate	0,5%	Solid petroleum jelly
11	Chlorpromazine hydrochloride	1%	Solid petroleum jelly
12	Thiourea	0,1%	Solid petroleum jelly
13	Lichen acid mix (atranorin, evernic acid, usnic acid)	0,3%	Solid petroleum jelly
14	Chlorhexidine	0,5%	Water
15	Paraphenylenediamine	1%	Solid petroleum jelly
16	*Compositae* mix (*Anthemis nobilis, Chamomilla recutita, Achillea millefolium Tanacetum vulgare Arnica montana*, parthenolide extract)	5%	Solid petroleum jelly
17	Ketoconazole	1%	Solid petroleum jelly
18	Diclofenac sodium	5%	Solid petroleum jelly
19	Piroxicam	1%	Solid petroleum jelly
20	Ethylhexyl methoxycinnamate	10%	Solid petroleum jelly

**Table 2:** UVB exposure according to phototype (Fitzpatrick).

Phototype	Dose (mJ)	Time of exposure (sec)
I	10‒20	10‒20
II	20‒30	20‒30
III	30‒40	30‒40
IV	40‒50	40‒50
V	50‒60	50‒60
VI	60‒70	60‒70

All patients were male, aged between 46 and 80 years old (mean of 61 years), 12 (57%) phototypes II‒IV and nine (43%) V and VI. The time of evolution was 18 months to 20 years, with no personal or family history of atopy. Eleven (52%) were occupational cases, eight (38%) of which were workers in areas exposed to the sun (five masons, one gravedigger, one agricultural worker, and one farmer), and six (28.5%) were retired. The affected areas were the face and upper limbs in 11 (52%), the back of the hands in nine (43%), the neck and upper chest region in eight (38%), the eyelids in three (14%), the lower limbs, abdomen or disseminated in two (9.5%), and the dorsum of the feet in one case (4.7%; Table 3).

**Table 3:** MOAHLFA index of the present study.

MOAHLFA	Number of patients ‒ n (%)
Male	21 (100%)
Occupational	11 (52%)
Atopic dermatitis	0
Hand dermatitis	9 (43%)
Leg dermatitis	2 (9,5%)
Facial dermatitis	11 (52%)
Age > 40 years	20 (95%)

Note: The MOAHLFA index is an acronym in English for the description of data on: male, occupational dermatitis, atopic dermatitis, hand dermatitis, leg dermatitis, facial dermatitis, and age >40 years, used to compare different populations undergoing patch testing.

Of the sixteen photo tests performed with UVB, two showed MED below their phototype. None of the patients showed sensitivity to UVA radiation.

Twelve patients (57%) had delayed hypersensitivity reactions to allergens from the standard battery and/or photo test battery, and nine (43%) tests were negative. Four cases (19%) had positive tests for three or more allergens. The detected allergens were: potassium bichromate in four patients (19%), balsam of Peru, nickel sulfate, and perfume mix, each present in three patients (14%), sesquiterpene lactone mix and *Compositae* mix, in two patients (9.5%) for each allergen, and BHT (Butylhydroxytoluene), PABA (Para-aminobenzoic acid), Paraphenylenediamine (PPDA), chlorpromazine, promethazine, epoxy resin, carba mix, Kathon CG, cobalt chloride, lichen acid mix in only one patient each, respectively (4.7%).

In the photo epicutaneous tests, 14 patients (66.6%) were positive, of which nine (38%) had concomitant delayed hypersensitivity to the assessed allergens, four (19%) were negative and three (14%) did not undergo the test. The identified photo allergens were: chlorpromazine, in five patients (24%), perfume mix, in four (19%), potassium bichromate and oxybenzone in three (14%), promethazine and *Compositae* mix in two (9.5%), BHT, paraphenylenediamine and sesquiterpene mix in one patient (4.7%).

CAD is common in temperate climates and in elderly Caucasian men, as observed in the present series. However, it has been reported in other regions and in young women with high phototypes.^1^ Associated with sun exposure, it may be occupational, as in 38% of the present series. None of the patients had a personal or family history of atopy or was HIV-positive. It has been described as having a variable period of evolution, which in the present series varied between 18 months and 20 years (an average of four years). It has a subacute and chronic course and initially presents with exuberant erythema and edema in sun-exposed areas, simulating erythroderma, but sparing covered areas. It develops into infiltrated and lichenified plaques and papules, affecting other areas such as the scalp and palmoplantar areas, and may have intense pruritus, generalized lymphadenopathy, onychodystrophy, and secondary diffuse alopecia. The infiltration, accentuating skin folds on the face, gives the patient a leonine facies. Two cases in the present series had erythroderma and the face and upper limbs were the most affected areas in eleven patients.^3^

A reduction in MED for UVA and UVB indicates more severe disease. A reduction for UVB only indicates mild to moderate conditions and is less frequent; it is more common for UVA only. All of the patients in the present series reported exacerbation or flare-up of the condition after sun exposure; however, only two of sixteen patients showed a reduction for UVB. Phototests are rarely normal, usually occurring at the onset of the condition. Ethnic and regional differences may explain these singularities, but there is a scarcity of publications on the geographic region assessed in the present series.^1^, ^2^

Approximately 70% of these patients may have allergic contact dermatitis or allergic contact photodermatitis.^4^ Of the present patients, 57% were positive for allergens; 66.6% for photoallergens, and 38% for both. Of these, 19% were positive for potassium bichromate (24% were masons), and 19% had three or more positive results.

Paraphenylenediamine, described as an emerging allergen due to the frequency of its use among women who dye their hair, may have a cross-reaction with sesquiterpene lactone, although they are not chemically related.^2^

Of the patients described in the present series, only one showed positivity to paraphenylenediamine, without relevance. Two showed sensitization to sesquiterpene lactone and *Compositae* mix, which are correlated, but not to paraphenylenediamine.

Recently, other cross-reactions have been reported between fragrance mix and colophonium, rubber allergens and metals (nickel and cobalt); the latter is more related to atopy.

The photo allergens most often associated with CAD are plants (sesquiterpene lactone), fragrances, ketoprofen and sunscreen compounds (avobenzone).^1^ In contrast, in the present study, the most frequently detected photoallergen was chlorpromazine (24%), which has analogs (dihydrochlorothiazide and promethazine) widely used in Brazil. Oxybenzone was found in 14% of cases, and sesquiterpene lactone in 4.7%.

The authors describe a series of patients with CAD whose demographic data and frequency of allergens and photo allergens differ from other geographic regions. The authors emphasize the importance of epicutaneous and photo epicutaneous tests for the diagnosis and management of these cases.

## Authors' contributions

Maria Antonieta Rios Scherrer: Design and planning of the study; data collection, or analysis and interpretation of data; statistical analysis; drafting and editing of the manuscript or critical review of important intellectual content; collection, analysis and interpretation of data; effective participation in research orientation; intellectual participation in the propaedeutic and/or therapeutic conduct of the studied cases; critical review of the literature; approval of the final version of the manuscript.

Mariana David Cangussu Fernandes Ribeiro: Drafting and editing of the manuscript; collection, analysis and interpretation of data; critical review of the literature; approval of the final version of the manuscript.

Hannah Barbosa Lopes dos Anjos: Drafting and editing of the manuscript; collection, analysis and interpretation of data; critical review of the literature; approval of the final version of the manuscript.

Vanessa Barreto Rocha: Design and planning of the study; critical review of important intellectual content; collection, analysis and interpretation of data; approval of the final version of the manuscript, effective participation in research orientation.

## Financial support

None declared.

## Conflicts of interest

None declared.
